# An evaluation study of a gender-specific smoking cessation program to help Hong Kong Chinese women quit smoking

**DOI:** 10.1186/s12889-015-2326-9

**Published:** 2015-09-29

**Authors:** Ho Cheung William Li, Sophia Siu Chee Chan, Zoe Siu Fung Wan, Man Ping Wang, Tai Hing Lam

**Affiliations:** School of Nursing, The University of Hong Kong, 4/F, William M. W. Mong Block, 21 Sassoon Road, Pokfulam, Hong Kong, China; School of Public Health, The University of Hong Kong, Hong Kong, China

**Keywords:** Chinese, Counselling, Gender-specific, Smoking cessation program, Women smokers

## Abstract

**Background:**

There is a lack of population-based smoking cessation interventions targeting woman smokers in Hong Kong, and in Asia generally. This study aimed to evaluate the effectiveness of a gender-specific smoking cessation program for female smokers in Hong Kong.

**Methods:**

To evaluate the effectiveness of the service, a total of 457 eligible smokers were recruited. After the baseline questionnaire had been completed, a cessation counseling intervention was given by a trained counselor according to the stage of readiness to quit. Self-reported seven-day point prevalence of abstinence and reduction of cigarette consumption (≥50 %) and self-efficacy in rejecting tobacco were documented at one week and at two, three and six months.

**Results:**

The 7-day point prevalence quit rate was 28.4 % (130/457), and 21.9 % (100/457) had reduced their cigarette consumption by at least 50 % at the six-month follow-up. The average daily cigarette consumption was reduced from 8.3 at baseline to 6.3 at six months. Moreover, both internal and external stimuli of anti-smoking self-efficacy increased from baseline to six months.

**Conclusions:**

The study provides some evidence for the effectiveness of the gender-specific smoking cessation program for female smokers. Furthermore, helping smokers to improve their self-efficacy in resisting both internal and external stimuli of tobacco use can be a way of enhancing the effectiveness of a smoking cessation intervention.

## Background

Cigarette smoking is the most important preventable cause of death and disease, causing six million deaths annually worldwide [1]. Previous studies have shown that half those who continue to smoke will be killed by tobacco prematurely [2, 3]. There is more evidence from the UK, US and Australia [4–8] to show that the habit could kill two thirds of smokers, especially those who started at a young age.

The current global population of woman smokers is far less than that of men [1]. However, while the epidemic of tobacco use among men is in slow decline, there is growing concern about increasing tobacco use in women [9, 10]. Smoking causes many fatal diseases and presents a major health threat to women [11, 12]. Some health consequences are specific to women, such as a higher rate of infertility, premature labor, low birth-weight infants, ectopic pregnancy, sudden infant death syndrome, cervical cancer, irregular menstruation cycles, dysmenorrhea and early menopause [11, 13]. As the health consequences of smoking only become fully evident 40 to 50 years after it was first taken up, the full effect on women’s health will only be seen after several decades. It is expected that the number of deaths among women attributable to tobacco will increase.

Hong Kong is the most Westernized and urbanized city of China. The prevalence of daily cigarette smoking among those aged 15 or older in Hong Kong decreased steadily from 23.3 % in 1982 to 10.7 % (19.1 % in men and 3.1 % in women) in 2012 [14]. Despite the general low prevalence of smoking, the 96,800 women who are daily smokers in Hong Kong cannot be overlooked or ignored [14]. Of them, about half have not attempted or do not want to quit, and 40.0 % have tried but failed [14]. However, while women are still much less likely to smoke than men, their numbers have risen by 72.5 % since 1990, with the increasing population - from 56,100 to 96,800, reflecting an alarming situation. Most importantly, the tobacco industry is actively seeking new customers to replace those who have already quit smoking or who will die prematurely [15, 16].

A previous study [17, 18] found that the psychological and social factors leading Chinese women to start and continue smoking were complex, and that they might encounter more difficulty and have less confidence in quitting than men. The 2013 Thematic Household Survey Report by the Hong Kong Census and Statistics Department [14] highlights the fact that negative emotions and stress are important factors in both smoking initiation and continued tobacco use on the part of female smokers. Indeed, with the rapid changes in social and economic structures in Hong Kong over recent years, more women are joining the workforce. At the same time these women might encounter more difficulty, negative emotions and additional stress in balancing their busy family and working lives [19]. Therefore, for female smokers with such problems, it is vital that healthcare professionals focus on helping them understand the negative health consequences of smoking, and at the same time counseling them about alternative strategies for coping with negative emotions and stress. Apart from that, a small proportion of female smokers emphasized the societal pressure to be slim, and considered smoking to be a weight control strategy, fearing a gain in weight if they gave up [18]. It is crucial for healthcare professionals to correct the myths that smoking can regulate mood disorders or help to control weight. Nevertheless, there is a lack of population-based smoking cessation interventions targeting woman smokers in Hong Kong, and in Asia generally. No evaluation study on such interventions or services designed specifically for women has been reported. To reduce female smoking and promote cessation, it is important to design tailor-made interventions to communicate clearly to women the risks, especially the specific and additional risks to women, of continued smoking, and to motivate them to quit. The first smoking cessation hotline for female smokers in Hong Kong was established in 2006 by the School of Nursing and School of Public Health at the University of Hong Kong, with the aim of providing a gender-specific smoking cessation program for female smokers. This study aimed to evaluate the effectiveness of such program.

### Theoretical framework

The intervention reported here was guided by the Transtheoretical Model of Behavior Change (TTM) [20]. Prochaska and DiClementte [21] identified various stages and processes of self-change in smoking that corresponded to the five stages of change in the TTM: (1) current smokers who are not seriously considering quitting within the next six months (pre-contemplation); (2) current smokers who are seriously considering quitting within the next six months, but not within the next 30 days, and have not made a 24-h quit attempt in the past year (contemplation); (3) current smokers who are seriously considering quitting within the next 30 days and have made a 24-h attempt in the past year (preparation); (4) ex-smokers who have achieved total abstinence from one day to six months (action); and (5) ex-smokers who have achieved total abstinence for six months or more (maintenance). Prochaska and DiClementte [21] pointed out that individuals differed at each stage of readiness to change their behavior, and interventions should therefore be tailor-made according to the individual’s stage of change.

## Methods

### Design

The project had three phases: the first was to build up a network, Women Against Tobacco Taskforce (WATT), with 14 women’s organizations mobilizing the community to support smoking cessation among female smokers, conduct a needs assessment survey to ascertain the learning needs, knowledge, attitudes and practice of tobacco control and smoking cessation, and identify those interested in joining the training program; the second phase was to develop a smoking cessation training curriculum and deliver a workshop for woman volunteers to equip them with knowledge and skills in smoking cessation and build a rapport with WATT members; the third phase was to set up a hotline service to deliver a gender-specific smoking cessation program to female smokers in Hong Kong. To examine the effectiveness of this program, a one-group pre-test and repeated post-test, within subjects design was used.

### Participants

Female smokers referred by WATT and met the inclusion criteria were invited to participate in this study. The inclusion criteria were (a) female Hong Kong Chinese current smokers, (b) aged 15 years or above, (c) able to speak and understand Cantonese and (d) willing to receive face-to-face or telephone counseling. We excluded those who were participating in other smoking cessation programs or services.

### Training and counseling service

Based on the results of the needs assessment survey, we designed a tailor-made smoking cessation counseling training program for the woman volunteers. A one-day workshop for WATT affiliates was organized. The curriculum was specifically designed to recognize the characteristics of women who smoked and to instruct the volunteers in the psychological and behavioral therapies involved in managing them. Throughout the training, a variety of topics were covered, such as how to assess smoking status and stage of readiness to change, nicotine addiction and the provision of brief individualized advice and motivation to promote cessation, using the ‘Five As’ approach, which refers to (1) asking about tobacco use; (2) advising quitting; (3) assessing willingness to quit; (4) assisting in the quit attempt and (5) arranging follow-up. At the end of the program, the volunteers were capable of delivering sound cessation advice for women.

Prior to the intervention, female smokers received brief advice on smoking cessation given by the trained woman volunteers from the WATT. The volunteers were encouraged to raise awareness of the hazards of smoking to women’s health and the importance of smoking cessation, provide brief cessation advice to female smokers according to their needs in their respective communities, and refer them to receive gender-specific smoking cessation intervention

### Gender-specific smoking cessation intervention

The intervention was delivered by female registered nurses who had attended a smoking cessation counseling program organized by the School of Nursing at the University of Hong Kong and had been awarded a certificate as a smoking cessation counselor after passing an examination.

During the intervention, the counselor first assessed the subject’s smoking and quitting history, and designed an individualized quit plan for her at the baseline interview. An intervention was then given by the counselor according to the subject’s stage of readiness. In the case of smokers at the pre-contemplation stage, counselors would increase their awareness of the need to quit smoking. For those at the contemplation stage, counselors would motivate them and enhance their confidence in the ability to quit, and reinforce their achievement in previous quit attempts. For those at the preparation stage, counselors would boost their self-efficacy in resisting smoking and discuss possible withdrawal symptoms. The counseling also included an explanation of the adverse effects of smoking on women’s health, identifying the barriers and facilitators to quitting, working with the subject to design an individualized quit plan, and teaching some relapse prevention strategies. In the case of smokers who had a concern about weight gain if they quitted smoking, counselors would correct the myths that smoking can control weight, and at the same time counselors would discuss and advise them about alternative strategies for weight control. Such an intervention was also given to subjects at the one-month follow–up, which aimed to assess the progress of the action plan and the barriers encountered in the behavioral change process, as well as to engage them in that process, enhance their self-efficacy and identify individual barriers and facilitators.

A mechanism was set up to assure the quality of the counseling interventions. First, the research team held regular meetings with the counselors every two months to discuss cases and evaluate the counseling. Second, the nurse counselors audio-taped one session per month and completed a self-assessment form for audit checking purposes. Before audio-taping, participants were well informed and verbal consents were obtained. An experienced nurse supervisor reviewed the audio tapes and completed a performance assessment form for cross-validation.

### Measures

#### Demographics and smoking characteristics

Baseline data including demographic and socio-economic characteristics and smoking history were obtained from each subject using a structured questionnaire, administered by a trained female nurse counsellor. The content of the structured questionnaire included smoking related information such as daily cigarette consumption, nicotine dependency assessed by the Fagerstrom test [22], stage of readiness to quit according to TTM [23] and previous quit attempts. Moreover, subjects’ psychological perspectives on behavior changes were also investigated by asking their perception on the importance, confidence and difficulty in quitting smoking on a scale of 0 to 100, with higher score indicating more.

#### Smoking Self-efficacy Questionnaire (SEQ-12)

Subjects’ self-efficacy against tobacco was assessed by using by the SEQ-12 [24]. The SEQ-12 is categorized into two subscales, namely internal stimuli (6 items) and external stimuli (6 items), with total possible scores ranging from 6 to 30 for both internal stimuli and external stimuli. Higher scores of the SEQ-12 on both subscales indicate greater self-efficacy to refrain from smoking. The SEQ-12 measures confidence in ability to refrain from smoking when facing internal stimuli (e.g. feeling nervous) and external stimuli (e.g. being with smokers). The psychometric properties of this scale have been empirically examined with the Cronbach's alpha coefficients of 0.95 and 0.94 for internal stimuli and external stimuli, respectively, indicating excellent internal consistency [25]. The intraclass correlation coefficients were 0.95 and 0.93 for internal stimuli and external stimuli, respectively, demonstrating excellent test-retest reliability [25].

The primary outcome measure at the six-month follow-up was the self-reported 7-day point prevalence of abstinence. Secondary outcomes included: (1) self-reported reduction of ≥ 50 % in cigarette consumption, and (2) self-efficacy against smoking at 6 months. Other outcomes were also assessed, examining factors that predicted successful quitting or a reduction in cigarette consumption after six months.

#### Data collection

This study was approved by Institutional Review Board of the University of Hong Kong and Hospital Authority Hong Kong West Cluster (reference UW 06–323 T/1348). Female smokers were recruited through WATT members’ referrals. Those eligible were invited to participate in the study after they were told its purpose. They were given the option of participating or refusing and were told that their participation was voluntary without prejudice. To introduce greater flexibility as subjects received the tailor-made intervention, they were able to select either a face-to-face or a telephone intervention at baseline and one month. Written consent was obtained from those subjects electing face-to-face counseling at baseline and verbal consent from those receiving telephone counseling at baseline. In the case of those under 18, written informed consent was obtained from their parents or guardians. After consent was obtained, the subjects’ smoking status and quitting history were assessed by the nurse counselors. In addition, this information was collected at one-week and one-, three- and six-month follow-ups. Continuous assessment allowed the counselors to monitor participants’ quitting processes and provide further reinforcement of behavioral changes.

#### Data analysis

Data analysis was performed using the Statistical Package for Social Science software, version 20.0 for Windows. Descriptive statistics were used to calculate the frequency and percentage (categorical data) or the mean and standard deviations (continuous data) of different demographic and social-economic characteristics. Chi-square test was used to detect any difference in self-reported 7-day point prevalence quit rate at 6-month follow up between those who received face-to-face counseling and those who received telephone counseling. According to our previous experiences in conducting smoking cessation interventions, most participants who were lost to follow-up or refused to further participate were people who had relapse or resumed smoking. Therefore, intention-to-treat analysis was used, with participants who lost to follow-up treated as smokers with no reduction in cigarette consumption compared with baseline. Paired *t*-test and chi-square test were used to compare data between baseline and 6-month follow-up for those who continued smoking. Bi-variate analysis was used to examine associations between variables at baseline and self-report tobacco abstinence in the past 7 days at 6-month follow up. Logistic regression analyses were conducted to identify predictors of successful quitting and reduction in cigarette consumption by at least 50 % at 6-month follow-up, with female smokers who continued to smoke as the reference group. The technique of backward elimination was used to minimize suppressor effect, which ensured that a variable could only make statistically significant contribution when other variables were controlled or held constant.

## Results

### Subject recruitment and retention rates

**Fig. 1:**
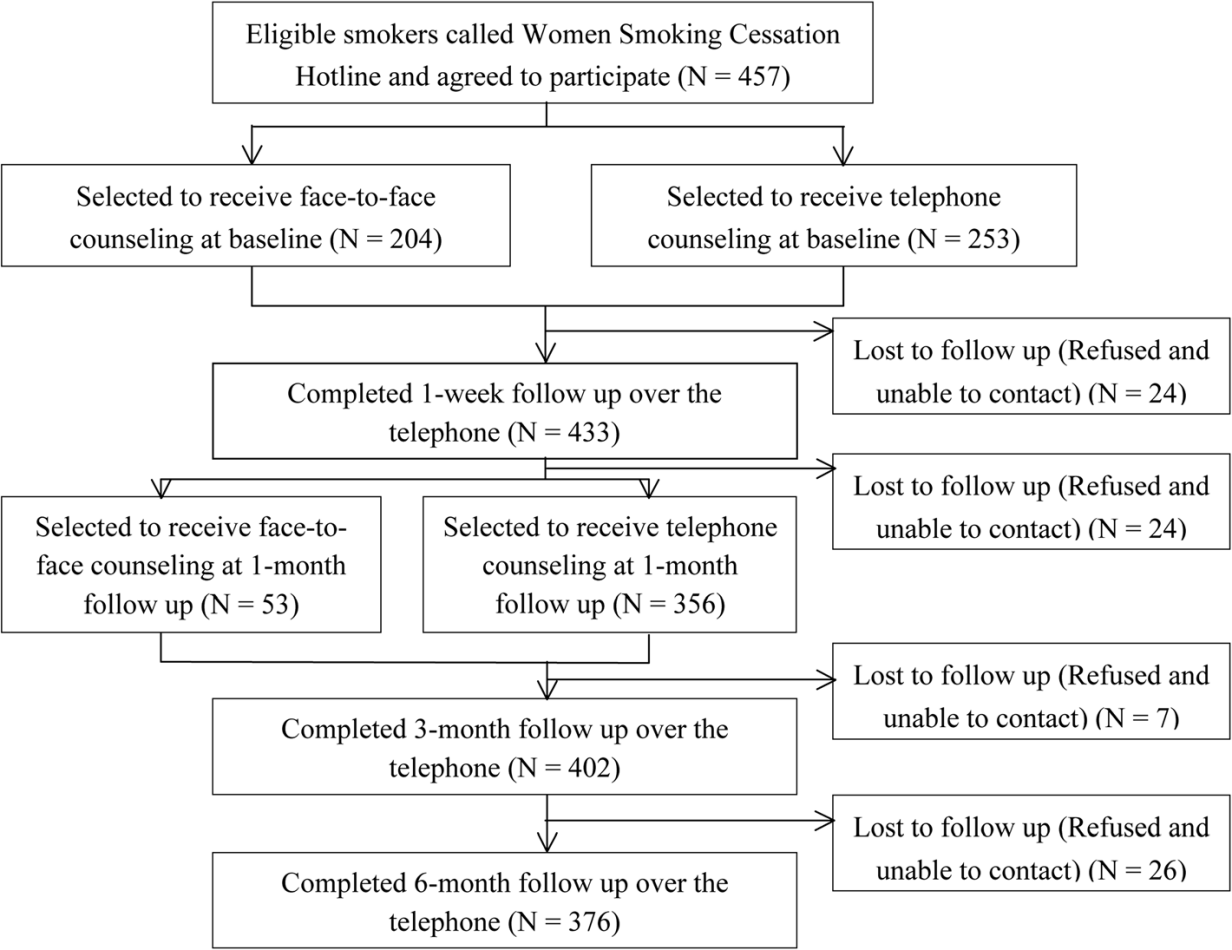
Flow chart of subject recruitment and retention rates

We received 895 phone calls, and a total of 457 eligible female smokers were recruited from November 2006 to March 2012. The recruitment and retention rates are shown in Fig. 1. The retention rates at follow-ups were 94.7 % (433/457) at one week, 89.5 % (409/457) at one month, 88.0 % (402/457) at three months and 82.3 % (376/457) at six months. A comparative analysis was performed to compare the demographic and baseline characteristics between those who completed and lost to follow-up at 6 month of the study. There were no statistically significant differences in any of the demographic and baseline data between those who completed the study and those who lost to follow-up at 6 month. Of the 457 subjects, 44.6 % (*n* = 204) chose face-to-face counseling at baseline, while 55.4 % (*n* = 253) elected telephone counseling.

### Demographic and socio-economic characteristics and psychological perspectives of the subjects

Table 1 shows that the mean age of the subjects was 34.8, 43.5 % were single, more than 90 % had attained secondary education or above, and 69.1 % were employed. On average, they had started smoking at 18.1 years and smoked 13.5 cigarettes daily. About a third had severe, moderate and mild levels of nicotine dependency, respectively. The majority (71.1 %) had already made at least one quit attempt. The mean perceived importance, difficulty and confidence scores in quitting smoking were 86.5, 74.9 and 60.1 (out of 100), respectively. The internal and external stimuli to self-efficacy against smoking were 13.3 and 15.6, respectively. The results showed that there was no statistically significant difference in the self-reported seven-day point prevalence quit rate at the six-month follow-up between those who received face-to-face counseling and those who chose telephone counseling.

**Table 1:** Characteristics of participants at baseline (*N* = 457)

Characteristics	*N* (%)/Mean ± SD	
Demographics:		
Age^a^	34.8 ± 9.8	
Marital status^b^		
- Single	198	(43.5)
- Married	183	(40.2)
- Cohabiting, divorced, separated and widowed	74	(16.3)
Educational attainment^c^		
- No formal education	4	(0.9)
- Primary school	22	(4.9)
- Secondary school	329	(72.6)
- Post-secondary, tertiary or above	98	(21.6)
Occupational status^d^		
- Employed	311	(69.1)
- Housewife	89	(19.8)
- Unemployed	35	(7.8)
- Retired	7	(1.6)
- Full-time student	8	(1.8)
Tobacco use related:		
Daily cigarette consumption	13.5 ± 7.8	
Age started smoking	18.1 ± 5.7	
Nicotine dependency level by the Fagerstrom test (0–10)		
- Mild (0–3)	177	(38.7)
- Moderate (4–5)	147	(32.2)
- Severe (6–10)	133	(29.1)
Other smokers in the same household^b^		
- Nil	288	(63.3)
- One or more	167	(36.7)
Smoking status of spouse^e^		
- No spouse/ spouse is not smoker	314	(69.5)
- Spouse is smoker	138	(30.5)
Quitting History:		
Previous quitting attempt(s)^f^		
- Nil	129	(28.9)
- once	140	(31.3)
- 2 to 5 times	153	(34.2)
- 6 to 10 times	18	(4.0)
- more than 10 times	7	(1.6)
Psychological factors:		
Perceived importance on quitting (0–100)	86.5 ± 15.4	
Perceived difficulties on quitting (0–100)	74.9 ± 19.5	
Perceived confidence on quitting (0–100)	60.1 ± 22.0	
Self-efficacy against smoking^b^		
- Internal stimuli (6–30)	13.3 ± 3.6	
- External stimuli (6–30)	15.6 ± 3.6	

^a^Total *n* = 456, 1 missing; ^b^Total *n* = 455, 2 missing; ^c^Total *n* = 453, 4 missing; ^d^Total *n* = 450, 7 missing; ^e^Total *n* = 452, 5 missing; ^f^Total *n* = 447, 10 missing

Table 2 shows quitting or reduction in cigarette consumption at one week, one month, three months and six months. At the six-month follow-up, the seven-day point prevalence quit rate was 28.4 % (130/457), about 21.9 % (100/457) had reduced their cigarette consumption by at least 50 %, and the prevalence of quitting or reduction was 50.3 % (230/457). There were 11.4 % (52/457), 8.1 % (37/457) and 3.9 % (18/457) smokers who reduced their daily cigarette consumption by at least 50 % at 1 week, 1 month and 3 month, respectively quitted smoking at 6 months. Table 3 shows the smoking profile and psychological factors of those who continued to smoke between baseline and six-month follow-up. There were statistically significant differences in daily cigarette consumption and internal and external stimuli to anti-smoking self-efficacy, with the average daily cigarette consumption reduced from 8.3 at baseline to 6.3 at six months. Moreover, both internal and external stimuli to self-efficacy against smoking were increased from baseline to six-month follow-up.

**Table 2:** Quitting and reduction in cigarette consumption at 1 week, 1 month, 3 months and 6 months (*N* =457)

Characteristics	*n* (%)			
	1-week	1-month	3-month	6-month
Self-reported 7-day point prevalence quit rate	63 (13.8 %)	100 (21.9 %)	124 (27.1 %)	130 (28.4 %)
Had not quit but had reduced daily cigarette consumption by at least 50 %	197 (43.1 %)	165 (36.1 %)	137 (30.0 %)	100 (21.9 %)
Quit or had reduced daily cigarette consumption by at least 50 %	260 (56.9 %)	265 (58.0 %)	261 (57.1 %)	230 (50.3 %)

Intention-to-treat analysis was used

**Table 3:** The smoking profile and psychological factors for those who continued to smoke between baseline and 6-month follow up

	Baseline	6-month follow up	*p*-value
Daily cigarette consumption^a^	8.3 ± 0.5	6.3 ± 0.4	<0.001^*^
Self-efficacy against smoking			
- Internal stimuli^b^	12.6 ± 3.4	14.2 ± 4.0	<0.001^*^
- External stimuli^b^	15.3 ± 3.3	16.2 ± 3.5	0.001^*^

^a^Total *n* = 239, 7 missing; ^b^Total *n* = 241, 5 missing; *paired *t*-test

Bi-variate analysis showed that six factors were significantly associated with quitting or reduction in cigarette consumption: marital status, daily consumption, level of nicotine dependency, smoking status of spouse, and internal and external stimuli to self-efficacy against smoking. A logistic regression model to predict successful quitting at six months is shown in Table 4. The results showed that being married (odds ratio 1.86, 95 % CI 1.20–2.86), and having a lower daily cigarette consumption and a higher self-efficacy against internal stimuli (odds ratio 1.10, CI 1.03–1.17) were predictors of successful quitting. A second logistic regression model, to predict reduction in cigarette consumption by at least 50 %, appears in Table 5. The results showed that a lower daily cigarette consumption (odds ratio 0.95, 95 % CI 0.92–0.97), and a mild (odds ratio 1.95, CI 1.14–3.32) to moderate (odds ratio 1.82, CI 1.05–3.18) level of nicotine dependency were predictors of a reduction in consumption of at least 50 %.

**Table 4:** Logistic regression (backward) model to predict successful quitting at 6-month follow-up

Independent variables	OR (95 % CI)	*p*-value
Married	1.86 (1.20–2.86)	0.005
Daily cigarette consumption	0.94 (0.91–0.98)	0.001
Self-efficacy against internal stimuli of smoking	1.10 (1.03–1.17)	0.004

OR = adjusted odds ratio; CI = Confidence interval

**Table 5:** Logistic regression model to predict the reduction in cigarette consumption by at least 50 % at 6-month follow-up

Independent variables	OR (95 % CI)	*p*-value
Daily cigarette consumption	0.95 (0.92–0.97)	<0.001
Level of nicotine dependency Mild	1.95 (1.14–3.32)	0.004
Moderate	1.82 (1.05–3.18)	0.033

OR = adjusted odds ratio; CI = Confidence interval

## Discussion

The rates of smoking among women have greatly increased, but support for tobacco control in the population remains relatively limited. With the increased risks to women of smoking-related diseases and early mortality, promoting women’s smoking cessation and general health should be given a high priority in the community. Because the psychological and social factors causing women to start and continue smoking may be different from those in other populations [26], providing gender-specific interventions is crucial.

The hotline service described above is the first to offer tailor-made smoking cessation interventions to female smokers in Hong Kong. Most importantly, the establishment of WATT has created a community-based network to promote quitting among women smokers and to arouse public awareness of the hazardous effects of smoking, with an emphasis on women-specific problems and diseases. The counselors provide individualized advice tailored for women smokers, to improve their self-efficacy in resisting smoking and to deal with withdrawal symptoms so that they gain confidence and increase control over their own behavior. The overall results provide some support for the effectiveness of such interventions, as evidenced by a high quit rate (28.4 %) and a significant reduction in daily cigarette consumption among subjects who continued to smoke at the six-month follow-up. The quit rate here is comparable to that (27 %) in a local smoking cessation clinic with face-to-face counseling plus nicotine replacement therapy (NRT) [27]. The quit rate in the present study is also higher than that achieved by the Hong Kong public ‘Quitline’, with a rate of 12 % [28]. There are certain factors that may explain why the quit rate appears to be higher in this study than for other smoking cessation interventions among local services. First, all the nurse counselors in this study were female and equipped with a sound knowledge of local women’s smoking. A thorough understanding of woman smokers, especially the psychological and social factors causing them to start and continue smoking, is a necessary step toward designing individualized tailor-made interventions to reduce female smoking and promote cessation. Second, our service provides a flexible mode and schedule for counseling. Subjects may select either face-to-face or telephone counseling at baseline and one-month follow up. The telephone counselors accept the subject’s own schedule and provide counseling at flexible times from Monday to Sunday (9:00 a.m. to 9:00 p.m.). Additionally, although Hong Kong is the most highly Westernized city in Asia, people are still greatly influenced by Chinese culture. Traditionally, smoking is mostly seen as a man’s habit and women who smoke are identified as indecent, problematic and badly behaved [28]. Therefore, one advantage of using female counselors is that women may feel more comfortable, with less pressure and embarrassment, when using the service. A previous local study found that the hotline service encouraged people who were reluctant to seek help from smoking cessation clinics, and was more attractive to females than to the general smoking population [28].

Apart from a high quit rate, subjects showed improvement in their self-efficacy in resisting smoking and their confidence in quitting, with less perceived difficulty, after receiving the intervention. A previous study found that smokers who had greater self-efficacy against tobacco use were more likely to quit [29]. The findings of the present study have important implications for practice. Helping smokers to improve their self-efficacy in resisting both internal and external stimuli leading to tobacco use can be a way of enhancing the effectiveness of cessation interventions.

A backward logistic model identified being married, having a lower daily cigarette consumption and a higher self-efficacy against internal stimuli to using tobacco at baseline were significant independent predictors of quitting at the six-month follow-up. Our findings are consistent with those of two other studies in Asian countries [30, 31], which found that those who smoked fewer cigarettes per day and had higher self-efficacy were more likely to quit smoking. Being married was also a significant predictor of quitting in our study. Married subjects may have more support and/or pressure from the family than their single counterparts and thus a greater chance of successful quitting [32].

### Limitations

The service provided multi-session counseling (baseline, one-week and one-month) and telephone follow-up to female smokers. However, some participants were lost to follow-up as a result of changing cell-phone numbers, unanswered phone calls or a refusal to participate further. Around 20 % of the participants did not receive a complete intervention in this way, thus weakening the effectiveness of the service. Another limitation of the study was the lack of a control group. The effectiveness of our program could not be confirmed by comparing the quit rates of smokers who accepted counseling and those who did not. Although the results may be confounded by other factors, the 28.4 % cessation rate in this study is higher than an unassisted quit rate (about 7 %) in adults, suggesting the real-world effectiveness of a gender-specific smoking cessation hotline for women. Nevertheless, future research may consider conducting a randomized controlled trial, with one group of female smokers receiving gender-specific counseling and another receiving a non-gender-specific intervention. Additionally, although the self-reported quit rate in this study appears to be higher than those in other local services, the rates from different settings may not be comparable; and all the rates above, including ours, had no biochemical validation.

### Implications for practice

The findings from this project have important implications for practice. The project took a pioneering role in providing a gender-specific smoking cessation counseling service for female smokers in Hong Kong, and the results show that 50.3 % of subjects quit or reduced smoking. Their self-efficacy in resisting tobacco use also improved simultaneously. The findings provide support for a gender-specific smoking cessation hotline service for Hong Kong Chinese female smokers. In addition, the results show the feasibility of this community model promoting a gender-specific service for female smokers.

The running of the hotline service was supported by the Health Care and Promotion Fund to the amount of HK$300,000 (USD38840) for two years. The costs included training women volunteers, staffing and general expenses. We have collaborated and formed a network with local women’s organizations to promote quitting and to arouse public awareness of the hazardous effects of smoking among women. This project can enhance the community’s capacity to promote smoking cessation because the knowledge, attitude and practice on smoking cessation of the trained women volunteers would be enhanced significantly. Most importantly, the project is sustainable because the trained women volunteers can continue to deliver the smoking cessation message to the public after project completion. We continued the hotline service within the capacity at the School of Nursing through mobilizing our existing facilities and resources. However, it is vital that the Hong Kong government and others take the initiative to test-run similar programs, collaborate with women’s organizations, and increase resources for women-oriented smoking cessation services.

## Conclusions

Despite some potential limitations, this study addresses a gap in the literature by evaluating the effectiveness of a gender-specific smoking cessation hotline service for Hong Kong Chinese female smokers. The findings of the study suggest such a service is feasible and likely to increase quit rates.

## Abbreviations

CI: Confidence Interval
NRT: Nicotine Replacement Therapy
SEQ-12: Smoking Self-efficacy Questionnaire
TTM: Transtheoretical Model of Behavior Change
UK: United Kingdom
US: United States
WWATT: Women Against Tobacco Taskforce
